# Grape Seeds: Chromatographic Profile of Fatty Acids and Phenolic Compounds and Qualitative Analysis by FTIR-ATR Spectroscopy

**DOI:** 10.3390/foods9010010

**Published:** 2019-12-21

**Authors:** Massimo Lucarini, Alessandra Durazzo, Johannes Kiefer, Antonello Santini, Ginevra Lombardi-Boccia, Eliana B. Souto, Annalisa Romani, Anja Lampe, Stefano Ferrari Nicoli, Paolo Gabrielli, Noemi Bevilacqua, Margherita Campo, Massimo Morassut, Francesca Cecchini

**Affiliations:** 1 CREA-Research Centre for Food and Nutrition, Via Ardeatina 546, 00178 Roma, Italy; alessandra.durazzo@crea.gov.it (A.D.); g.lombardiboccia@crea.gov.it (G.L.-B.); stefano.nicoli@crea.gov.it (S.F.N.); paolo.gabrielli@crea.gov.it (P.G.); 2 Technische Thermodynamik, University of Bremen, Badgasteiner Str. 1, 28359 Bremen, Germany; jkiefer@uni-bremen.de (J.K.); anjalampe@uni-bremen.de (A.L.); 3 Department of Pharmacy, University of Napoli Federico II, Via D. Montesano 49, 80131 Napoli, Italy; 4 Department of Pharmaceutical Technology, Faculty of Pharmacy, University of Coimbra, 3000-548 Coimbra, Portugal; souto.eliana@gmail.com; 5 CEB-Centre of Biological Engineering, University of Minho, Campus de Gualtar, 4710-057 Braga, Portugal; 6 PHYTOLAB, University of Florence, 50019 Sesto Fiorentino (Firenze), Italy; annalisa.romani@unifi.it (A.R.); margherita.campo@unifi.it (M.C.); 7 CREA-Research Centre for Viticulture and Enology, 00049 Velletri (Roma), Italy; noemi.bevilacqua@crea.gov.it (N.B.); massimo.morassut@crea.gov.it (M.M.); francesca.cecchini@crea.gov.it (F.C.)

**Keywords:** grape, grape seeds, FTIR spectroscopy, chemometrics, fatty acids, phenolic compounds, biorefinery, nutraceuticals

## Abstract

The primary product of the oenological sector is wine. Nonetheless, the grape processing produces large amounts of by-products and wastes, e.g., the grape seeds. In the context of a sustainable production, there is a strong push towards reutilizing these by-products and waste for making useful derivatives since they are rich of bioactive substances with high additional value. As it is true for the wine itself, bringing these by-products derivatives to the market calls for quality measures and analytical tools to assess quality itself. One of the main objectives is to collect analytical data regarding bioactive compounds using potentially green techniques. In the present work, the profile of fatty acids and the main phenolic compounds were investigated by conventional methods. The qualitative analysis of the main functional groups was carried out by Fourier Transform Infrared (FTIR) spectroscopy. Moreover, the successful use of FTIR technique in combination with chemometric data analysis is shown to be a suitable analytical tool for discriminating the grape seeds. Grape seeds of different origin have different content of bioactive substances, making this technique useful when planning to recover a certain substance with specific potential application in health area as food supplement or nutraceutical. For example, Cesanese d’Affile seeds were found to have a rather high fat content with a significant fraction of unsaturated fatty acids. On the other hand, the seeds of Nero d’Avola exhibit the highest amount of phenolic compounds.

## 1. Introduction

The valorization of the agro-food waste represents an important goal for the preservation and support of a sustainable ecosystem and effective production. The new concept of circular economy applied to agricultural recycling perfectly fits to a modern “zero waste” lifestyle, and can be achieved by biorefineries, bioenergy plants, and environmentally friendly processes for the production of biomolecules on both small and large industrial scale [1,2,3,4]. Lucarini et al. [5] gave an overview of how by-products and wastes from the wine industry can be used as biorefinery feedstock.

The oenological sector’s main product is the wine, and, to some extent, non-alcoholic juices. However, the process of winemaking produces wastewater, pomace (the solid residues of grapes), and lees, that require disposal or beneficial use, if possible. The winemaking process generates a considerable amount of organic solid waste [6], e.g., during the crashing-pressing processes and the wine clarification. Concerning the vinification process, the white vinification (without maceration step of the grape skins in the must) directly produces stalks and pomace. On the other hand, the red vinification process (with the grape skin maceration step in the must) leads to the immediate formation of steams and, only after a period of maceration, the pomace. Both vinification processes produce lees after the decanting [7]. After the pressing for juice/must, the pomace contains mainly: (i) skins; (ii) seeds; (iii) pulp residues of the grape. At the end of the fermentation, the lees that are separated from the wine during the clarification and decanting process, consist mainly of dead yeasts [8,9].

As aforesaid, the grape seeds are a relevant part of the waste. For this reason, in the last decade an ever-increasing interest in the seeds appeared, since they contain bioactive compounds such as fatty acids and polyphenols [5,10,11,12,13,14], which are attractive from a nutraceutical perspective [15,16,17,18,19,20,21,22]. Their potential benefits ranges from anti-platelet and anticoagulant activity, to antioxidant, hypoglycemic, and even activity against cancer [23,24,25,26,27].

The extracts obtained from processing the grape seeds can be useful ingredients for agronomic, food, nutraceutical, cosmetic, and pharmaceutical derivatives. For example, the oils that can be extracted from grape seeds are of high value, as they contain large fractions of polyunsaturated fatty acids [28]. Consequently, they can be brought to the market at relatively high prices. Hence, there is the need of analytical methods to confirm the authenticity and quality of by-products and wastes in the oenological sector.

While chromatographic techniques are still seen as the gold standard for analytical purposes in the winemaking industry, the spectroscopic methods are nowadays being reconsidered. They often do not require sample preparation steps and they are faster. Moreover, they can virtually provide information about all species present in the sample in one single step experiment without the need for any preliminary separation step as happens for other analytical techniques. Fourier-transform infrared (FTIR) spectroscopy is a very promising tool in this context. For example, applied to wine it is capable of determining a multitude of parameters including the alcohol content, the total acidity, the sugar content, the pH value, as well as the relative density [29,30].

Grape seeds were also studied by FTIR spectroscopy. Ismail et al. [31] used FTIR to study and quantify bioactive compounds in grape seeds. They identified carboxylate groups from gallic acid and proanthocyanidin gallate in the aqueous seed extract. Mohansrinivasan et al. [32] used FTIR analysis to identify the functional groups of the bioactive compounds present in grapeseed extracts obtained from ethyl acetate, water, and petroleum ether. Canbay and Bardakçı [33] applied a hexane extraction and further processing to grape seeds in order to yield fatty acid methyl esters (FAME), which were subsequently analyzed by FTIR spectroscopy. Nogales–Bueno et al. [34] utilized near-infrared (NIR) hyperspectral tools for the screening of extractable polyphenols in red grape skins. In further studies of their group [35,36], FTIR and Raman spectroscopy were applied to grape seed samples. They were able to find correlations between the spectral features and the phenolic extractability as well as other attributes in the grape skin and grape seed. From their studies [34,35,36], Nogales–Bueno et al. concluded that FTIR spectroscopy coupled with chemometrics represents a valuable tool for monitoring the composition of wine by-products. Such analysis can be utilized to identify the most suitable extraction process. Further applications of FTIR in the oenological sector included the investigation of the biodegradation of winery and distillery wastes during composting [37] and the analysis of grape seed oils [38,39].

The project behind the present study has a wider scope in the context of a circular economy in the oenological sector. One of its main objectives is to collect analytical data relating to the bioactive compounds present in the waste using potentially green techniques. In this connection, the present paper aims at extending the use of FTIR spectroscopy in the oenological sector by demonstrating that the method can also be used to discriminate grape seeds between different cultivars. For this purpose, grape seeds from Cesanese d’Affile (Lazio, Italy), Greco bianco (Campania, Italy), and Nero d’Avola (Sicilia, Italy) were characterized for their fatty acid content and the main phenolic compounds. Then, the attenuated total reflection (ATR) FTIR technique was applied for the qualitative analysis of the functional groups present in the extract and a multivariate analysis was carried out for the authentication and discrimination of the samples.

## 2. Materials and Methods

### 2.1. Plant Materials

The study was carried out using 3 *Vitis vinifera* (L.) cultivars (white and red grapes), grown in an experimental field in the Lazio region (Italy) (41°40′12″ N latitude, 12°46′48″ E longitude) at 332 m above sea level in 2017. The vineyard was 17 years old. The grapes were harvested at technological maturation. The cultivars had a Cordon Spur training system with a plant density of 2.60 × 1.5 m. The same cultural practices were applied in the vineyard. Cesanese d’Affile [40,41] cultivar autochthonous of the Lazio region (Central Italy) was characterized by a medium compact cluster and of cylindrical shape with small berries of spherical shape, and grape seeds of medium size and an average number of 1–2 per berry. Nero d’Avola [42] was a cultivar autochthonous of the Sicilia region (South Italy) with a medium cluster and of cylindrical shape with of medium size, rather oblong, regular, intense black color and seeds of medium size and an average number of 2–3 per berry. Greco Bianco was a cultivar grown in the region of the central-southern Italy, with a medium compact cluster and of cylindrical shape with 1–2 wings, with berries of medium-small size with ellipsoidal shape, green-yellow color, and large seeds with an average number of 2–3 per berry.

The procedure of seeds separation was carried out as follows: from 10 grape clusters for each cultivar, 400 berries were randomly detached. The seeds from these berries were manually separated from the pulp. Then, they underwent a homogenization procedure to improve the reproducibility before the resulting substance was frozen at −30 °C and then lyophilized for the subsequent analysis. The lyophilization process guarantees the samples homogeneity and uniformity. In addition, this method allowed an optimal storage to protect the sample from oxidation and eventual possible contamination.

The lyophilized samples were ground in a refrigerated mill (Janke and Kunkec Ika Labortechnik, Germany) and the powder were sieved to obtain a granulometry of 0.5 mm.

### 2.2. Chemical Analysis

#### 2.2.1. Fatty Acid Analysis

Fat was extracted through the method of Bligh and Dyer [43]. An aliquot of the extract was used for gas chromatographic (GC) analysis. The fatty acids were esterified using 5% anhydrous hydrogen chloride in methanol as esterification reagent [44]. The esterified fatty acids were quantified by gas chromatograph (Agilent 7890A), equipped with both FID (Flame Ionization Detector) and MS (Mass Spectrometry, Agilent 5975C) detectors [45]. The separation of the fatty acids was accomplished on a Mega-wax column (30 m × 0.32 mm i.d., 0.25 µm film thickness). The GC system allows to acquire and record in the same injection both the FID and MS signals, for qualitative and quantitative determinations respectively. Identification was also carried out by comparing the retention time of detected compounds in the sample with those from a standard FAME mix (Supelco TM 37 component FAME mix C4-C24; Sigma-Aldrich, St. Louis, MO, USA). Quantification was performed calculating the internal percentage distribution of FAME.

#### 2.2.2. Phenolic Compound Analysis

For the extraction of phenolic compounds, the grape seeds were extracted with a solution EtOH:H_2_O 70:30 (pH 3.2 by addition of HCOOH), in a p/V ratio of 15%, under stirring for 24 h, then the extract was separated from the solid matrix by low pressure filtration.

The High-Performance Liquid Chromatography with Diode-Array Detection coupled with a Mass Spectrometer (HPLC/DAD/MS) analyses were performed with a HP 1100 liquid chromatograph equipped with a Diode Array (DAD) detector and a Mass Selective Detector (MSD) and with an Atmospheric Pressure Ionization API-electrospray (Agilent Technologies, Palo Alto, CA, USA). Mass spectrometer operating conditions were the following: gas temperature 350 °C at a flow rate of 10.0 L/min, nebulizer pressure 30 psi, quadrupole temperature 30 °C and capillary voltage 3500 V. The mass spectrometer operated in positive and negative ionization mode at 80–120 eV. The analytical column was a LiChrosorb RP18 250 × 4.60 mm, 5 µm (LichroCART, Merck Darmstadt, Germany) maintained at 26 °C. The eluents were H_2_O adjusted to pH 3.2 by HCOOH (A), and CH_3_CN (B). A 7-step linear solvent gradient system, starting from 100% A up to 100% B was applied during a 117-min period at a flow rate of 0.8 mL/min [46].

The phenolic compounds were identified by using data from HPLC/DAD/MS analyses, by comparing and combining their retention times, UV/Vis and mass spectra with those of the available specific commercial standards and according to the available literature data. All the solvents (HPLC grade) and formic acid (ACS reagent) were purchased from Aldrich Chemical Company Inc. (Milwaukee, WI, USA). The standards gallic acid and (+) catechin were purchased from Extrasynthèse S.A. (Lyon, Nord-Genay, France). Each compound was quantified by HPLC/DAD, using a five-point regression curve built with the available standards. Calibration curves with *r*^2^ ≥ 0.9998 were considered. All polyphenolic derivatives showed good linearity over the range tested with correlation coefficients *r*^2^ all above 0.9998.

The Limit of Detection (LOD) was obtained as the concentration corresponding to 3 times the noise recorded in the chromatograms; the Limit of Quantification (LOQ) was calculated as the concentration corresponding to 10 times the noise recorded in the chromatograms. The obtained values were: 0.21 µg/mL LOD and 0.68 µg/mL LOQ for catechin; 0.08 µg/mL LOD and 0.12 µg/mL LOQ for gallic acid. Gallic acid was calibrated at 280 nm using gallic acid as reference; catechin, epicatechin their oligomers were calibrated at 280 nm using (+) catechin as reference. In all cases, the actual concentrations of derivatives were calculated after making corrections for changes in molecular weight among compounds belonging to the same polyphenolic subclass.

#### 2.2.3. Statistical Analysis

All analyses were performed in triplicate. Data are presented as mean ± standard deviation (s.d.). Statistica for Windows (Statistical package; release 4.5; StatSoft Inc., Vigonza, PD, Italy) was used to perform One-way Analysis of Variance (ANOVA).

### 2.3. FTIR Analysis

The FTIR spectra were recorded on a Nicolet iS10 FT-IR spectrometer (Thermo-Fisher Scientific, Waltam, MA, USA) equipped with a diamond crystal cell for attenuated total reflection (ATR) operation. The spectra were acquired (32 scans per sample or background) in the range of 4000–500 cm^−1^ at a nominal resolution of 4 cm^−1^. The spectra were corrected using the background spectrum of air. The analysis was carried out at room temperature. For a measurement, a lyophilized sample was placed on the surface of the ATR crystal. Before acquiring a spectrum, the ATR crystal was carefully cleaned with wet cellulose tissue and dried using a flow of nitrogen gas. The cleaned crystal was checked spectrally to ensure that no residue was retained from the previous sample. For each sample, ten spectra were recorded. The spectrum of every sample was collected 10 times to check the reproducibility and do a statistical analysis. In addition to FTIR, the samples were analyzed conventionally to determine the fatty acid and phenolic compound profiles in order to aid the interpretation of the spectra, see next sub-sections for details.

The FTIR spectra were evaluated in two different ways: qualitative analysis of spectra and discrimination analysis.

#### 2.3.1. Qualitative Analysis of the Spectra

As a first step, they were analyzed with respect to the spectral band positions in order to identify the signatures of the major functional groups. An assignment of the main bands was carried out by analyzing the acquired spectra and by comparing them with the literature.

#### 2.3.2. Discrimination Analysis

In the second step, principal component analysis (PCA) was applied to the dataset. PCA is a statistical method that reduces the dimensionality of a data set by calculating the eigenvalue decomposition of the covariance matrix [47,48,49,50]. In other words, it identifies the spectral signatures that represent the variance of the data set. The results of a PCA are commonly discussed in terms of scores and loadings. The scores are the transformed variable values of a particular data point and the loadings represent the numbers by which each original variable should be multiplied to get the score. For a practical analysis, the scores and loadings plots are produced. The scores plot visualizes the scores with respect to the different principal components (PCs). A clustering of the data points in such a plot suggests that they exhibit spectral similarities and hence the corresponding samples can be assigned to a common category. The loadings of the individual PCs, on the other hand, can be plotted as a function wavenumber. The resulting spectra show characteristic signatures that allow a discrimination between the different categories. However, care must be taken when deciding how many PCs are to be considered. If a dataset’s variance is mainly represented by two PCs, the higher components are predominantly noise and, as a consequence, the results may be over-interpreted. The signal-to-noise ratio of the loadings plot is a good indicator to decide whether or not a PC should be included in the analysis.

In the present work, the PCA algorithm implemented in Matlab R2012 was used without initial data centering in order to keep the method as simple as possible.

## 3. Results and Discussion

This section first presents the fatty acid and phenolic compound profiles of the grape seeds in order to provide a better description/characterization of matrices as reference for the subsequent discussion of the spectroscopic data and their qualitative and multivariate analysis. In order to validate the FTIR technique as a routine method for the characterization of grape seed extracts, the different samples were analyzed using HPLC/DAD/MS and GC/MS methods to identify and quantify both phenolic compounds and fatty acids that represent the main compounds present in the samples, and detectable by FTIR. This could be the basis for the interpretation of some bands present in the FTIR spectra.

### 3.1. Chemical Analysis

#### 3.1.1. Fatty Acids

**Figure A1:**
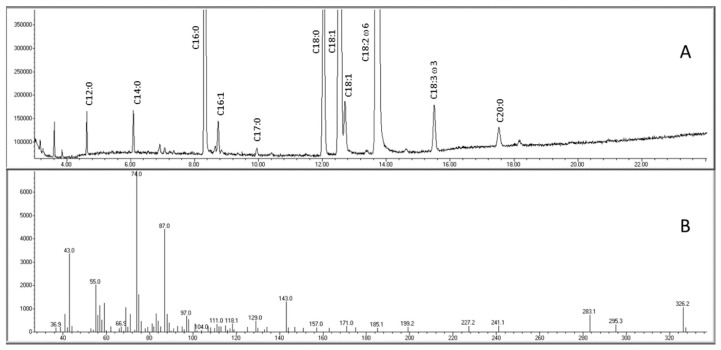
(**A**) Total Ion Chromatogram (TIC) of fatty acid profile of Nero d’Avola sample and (**B**) GC/MS spectrum used for peaks identification (example of arachidonic acid).

The fat content and the fatty acids profiles are summarized in Table 1 and an example chromatogram is shown in Figure A1. Cesanese d’Affile shows the highest value in fat content. It can be seen that the fatty acids profiles are reasonably similar within the margins of the measurement error. The main components are linoleic acid (C18:2 ω-6), oleic acid (C18:1), palmitic acid (C16:0), and stearic acid (C18:0). They add up to about 98% of the total fatty acids. Previous works have indicated grape seeds as a good source of the beneficial polyunsaturated fatty acids [51,52,53]. The current work of Pérez-Navarro et al. [51] reported a different profile of free fatty acids in the grape tissues, showing a higher proportion of unsaturated fatty acids in seeds (about 60%).

#### 3.1.2. Phenolic Compounds

**Figure A2:**
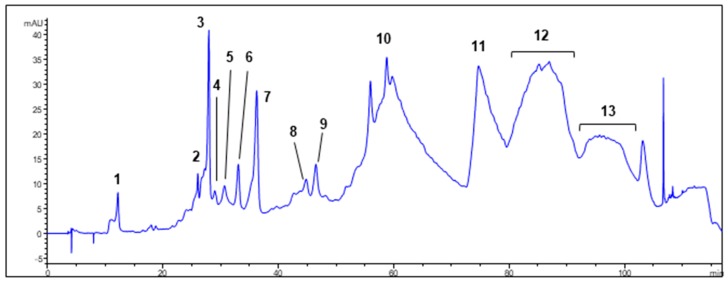
Chromatographic profile of Nero d’Avola seed extract, acquired at 280 nm. 1. Gallic acid; 2. Catechin dimer B3; 3. Catechin; 4. Procyanidin trimer; 5. Catechin dimer B6; 6. Catechin dimer B2; 7. Epicatechin; 8. Catechin trimer; 9. Epicatechin gallate dimer; 10. Catechin oligomers expressed as tetramers; 11. Epicatechin gallate dimer; 12. Catechin/epicatechin trimers digallate; 13. Catechin/epicatechin trimers gallate.

In Table 2 the main phenolic compounds present in grape seeds are summarized; a corresponding example chromatogram is shown in Figure A2. More distinct differences are observed for the phenolic compounds. The Nero D’Avola grape seed sample is rich both in flavan-3-ols (catechin and epicatechin) and their oligomeric or polymeric condensed derivatives (procyanidins). Instead, the Greco Bianco seeds exhibits the highest percentage of gallated compounds (35.78 mg/g on 46.70 total tannins, 76.6%) compared to Nero D’Avola (54.87 mg/g on 85.92 total tannins, 63.9%) and Cesanese d’Affile (39.32 mg/g on 57.80 total tannins, 68.0%). The highest weight oligomers, gallated trimers and tetramers, are more abundant in the Nero d’Avola sample (60.71 mg/g on 85.92 total tannins, 70.7%). Nero d’Avola seeds have also the highest content in total tannins and gallic acid (85.92 mg/g), followed by Cesanese d’Affile (57.80 mg/g) and Greco Bianco (46.70 mg/g). The results available in literature about grape seed extracts are not always consistent due to the different cultivars considered, areas of harvest, extraction techniques investigated and the use of solvents with different extraction capacities. Actually, grape seed extracts with variable titres from 15% up to 90% condensed tannins are available on the market for oenological, cosmetic or phytotherapic use, often lacking any information about characteristic of the raw material. In general, polyphenols and in particular condensed tannins and fatty acids are the most interesting and represented compounds in grape seeds [54,55,56]. The knowledge of the individual compounds and subclasses present in extracts with different polyphenolic contents has been used as a basis for the interpretation of some bands present in the FTIR spectra.

### 3.2. FTIR Data

#### 3.2.1. Qualitative Analysis of FTIR Spectra

**Figure 1:**
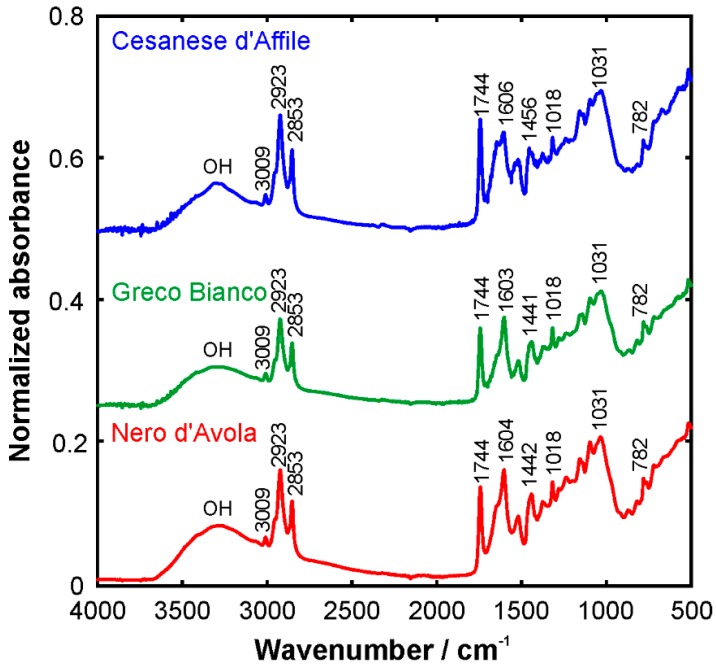
Averaged FTIR spectra of Cesanese d’Affile, Greco Bianco and Nero d’Avola grape seeds in the mid-infrared region (4000–500 cm^−1^).

FTIR provides a characteristic signature of the chemical or biochemical substances present in a sample by featuring their molecular vibrations (stretching, bending, and torsions of the chemical bonds) [57]. Therefore, the FTIR spectrum represents a molecular fingerprint of the sample. The averaged spectra from the grape seed samples are shown in Figure 1. It is possible to discern numerous peaks, which correspond to functional groups and modes of vibration of the individual components. The broad band peaking at around 3270 cm^−1^ corresponds to the OH stretching modes. It can be attribute to the polysaccharides and/or lignins as reported by [36,58,59]. The peak at 3009 cm^−1^ is related to the C-H stretching vibration of the cis-double bond (=CH) groups. Asymmetric and symmetric stretching vibrations of CH_2_ groups are found at 2923 and 2853 cm^−1^, respectively. They are mainly associated with the hydrocarbon chains of the lipids or lignins [54]. The spectral band at 1744 cm^−1^ and the shoulder band at 1716 cm^−1^ is attributed to the absorption of the C=O bonds of the ester groups and it is related to the presence of the fatty acids and their glycerides, as well as pectins and lignins [60,61]. The bands around 1600 cm^−1^ are associated with the stretching of C=OO^−^ and aromatic C=C groups, e.g., in pectins and phenolic compounds [61,62,63], but also with the bending vibrations of OH groups. The fingerprint region from 1500 to 800 cm^−1^ is very rich in peaks originating from various stretching, bending, rocking, scissoring, and torsional modes. This region is, on the one hand, very rich in information, but, on the other hand, difficult to analyze due to its complexity. This area provides important information about organic compounds, such as sugars, alcohols, and organic acids, present in the sample.

The aromatic C-C stretching at ~1520 and ~1443 cm^−1^ is related to phenolic compounds [58,59]. The CH_3_ out of plane bending at 1377 cm^−1^, the scissoring at 1318 cm^−1^, and the C-O stretching at ~1035 cm^−1^ are related to polysaccharide structures [58,61]. The peak at 1143 cm^−1^ corresponds to aromatic C-H stretching and the band at 782 cm^−1^ is due to the rocking of CH_2_, both in phenolic compounds [58].

#### 3.2.2. Multivariate Analysis of FTIR Spectra

**Figure 2:**
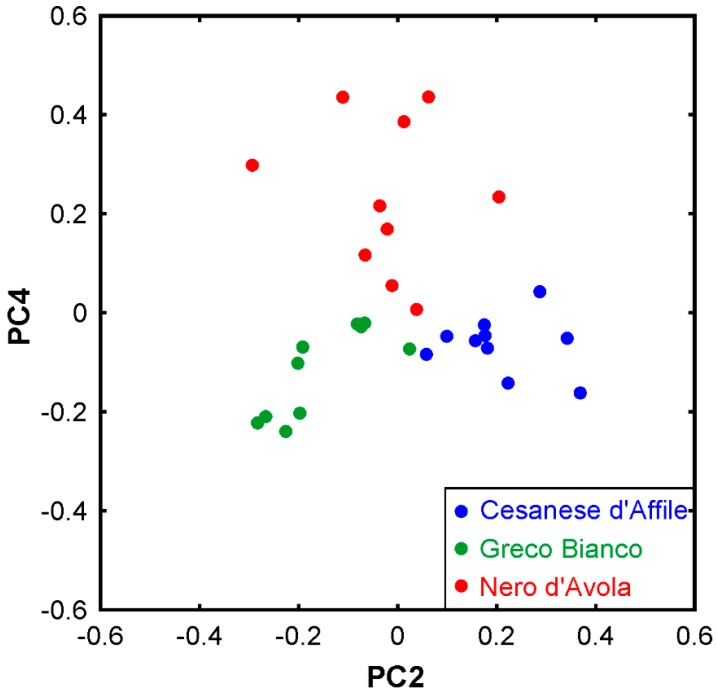
Score plot of the PCA of the FTIR spectra. Each dot represents the PC4 vs. PC2 scores of one spectrum recorded from an individual sample of grape seed.

**Figure A3:**
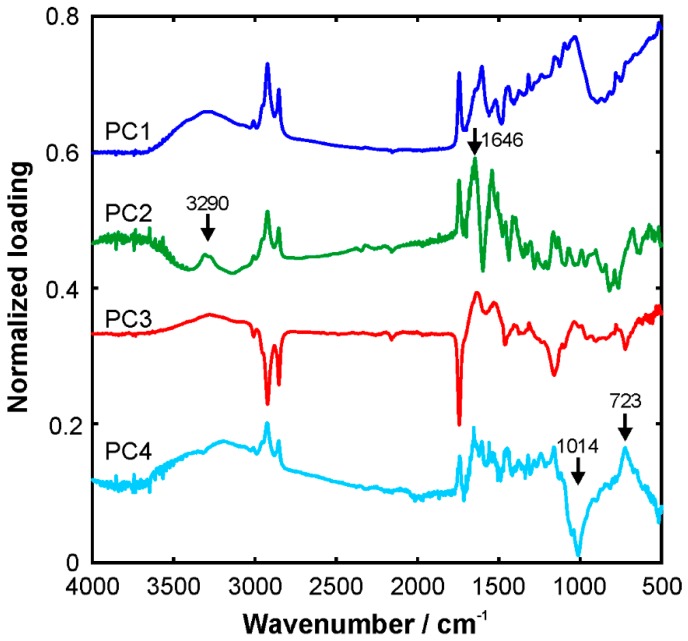
Loadings vs. wavenumber plot of the first 4 PCs of the FTIR spectra.

Overall, the spectra in Figure 1 appear very similar, which is reasonable due to the similarity in chemical composition. As we have seen in Section 3.1, the main differences appear in the phenolic compound profile, but the concentrations of these compounds are small. Nevertheless, small differences, e.g., in band shapes and relative intensities, can be observed in the spectra. In order to test, if an unsupervised classification of the individual spectra is possible, a PCA analysis of the full spectra was performed. This approach is commonly utilized for a classification of a data set. The first two principal components, PC1 and PC2, represent 99.4% of the variance of the data set. Out of this, PC1 accounts for 98.9%, which is in concert with the observation that the spectra appear very similar. In the score plot of PC1 and PC2, the seed from Cesanese d’Affile grape can already be distinguished from the Greco Bianco and Nero d’Avola seeds along the PC2 axis. Discriminating between the latter two requires further PCs. Therefore, Figure 2 presents the score plot of PC2 vs. PC4, in which all three grape seeds can be distinguished from each other. In this context, we note that PC3 and PC4 represent about 0.3 and 0.1% of the variance. This appears low at first glance, but their loadings vs. wavenumber plots exhibit relatively high signal-to-noise ratio with distinct spectral signatures. Therefore, utilizing them for the classification makes sense. For completeness, the normalized loadings plots are provided in the Appendix A, see Figure A3. We also note that applying the PCA to selected ranges of the spectra, e.g., the CH/OH stretching region, 2700–3700 cm^−1^, allowed discrimination with less components. However, selecting an appropriate range requires *a priori* knowledge and therefore was not further considered in the framework of this study. The same is true for the application of hierarchical cluster analysis (HCA), which was also implemented in Matlab to test its capability. Feeding the full spectra into the algorithm did not allow a sufficiently clear clustering. Therefore, this is not further discussed here.

When we have a closer look at the PCA discrimination described above, the analysis of the loadings plots in Figure A3 provides further insights. As aforesaid, Cesanese d’Affile can be distinguished from the other two along the PC2 axis. The peak at 1646 cm^−1^ is a dominating signature of this component. Even in the raw spectra (cf. Figure 1) there is a shoulder band in the data of Greco Bianco and Nero d’Avola seeds, while it appears a rather clear peak in the Cesanese d’Affile spectrum. The chemical analysis would suggest that this signature originates from the fats and fatty acids as Cesanese d’Affile exhibits a higher content. However, previous FTIR studies of oils and pure fatty acids show no peaks in this region at all [64]. Given the fact that there is another characteristic feature in the OH stretching region of the PC2 loadings spectrum, it is likely that both signatures originate from hydroxyl groups. The narrow bandwidth indicates that these OH groups are distinctly bonded to their molecular surroundings via hydrogen bonds. Unravelling the molecular phenomena further requires advanced computational approaches such as molecular dynamics simulations and quantum chemistry. This is beyond the scope of the present work.

Nero d’Avola can be distinguished from the others along the PC4 axis. The PC4 loadings spectrum reveals characteristic signatures at 723 and 1014 cm^−1^ as well as a broad band in the OH stretching region. They can all be attributed to phenolic compounds, which makes sense given that Nero d´Avola exhibits the highest content of this category.

## 4. Conclusions

This research showed that grape seeds are rich in beneficial polyunsaturated fatty acids and polyphenols and hence they can represent a promising source of nutraceuticals. The analysis of the chemical profiles of Cesanese d’ Affile, Greco bianco, and Nero d’Avola seeds revealed that Cesanese d’ Affile exhibits the highest fat content with a significant fraction of unsaturated fatty acids. On the other hand, the seeds of Nero D’Avola show the highest amount of phenolic compounds.

Moroever, we have shown that the grape seeds from different grape cultivars can be distinguished by FTIR-ATR spectroscopy using a chemometric data analysis. It was possible to link selected spectral signatures, which the principal component analysis picks up for the discrimination, with the chemical profiles. This is interesting as PCA is a purely mathematical tool, but to some extent it helps to understand the underlying physics. Overall, we conclude that FTIR spectroscopy is a suitable tool for applications in the oenological sector. This may facilitate the advanced detection of adulteration in the future. The term “advanced” in this context means that, e.g., the mixing of certain grape seeds with other “cheaper” ones from a minor quality cultivar can be revealed. In order to tap the full potential of FTIR spectroscopy it should be combined with multivariate data analysis. If the algorithms are trained with sufficiently large calibration data sets, such analyses can yield a multitude of parameters and make other (often more expensive and time consuming) analytical methods become redundant. Further FTIR/chemometrics studies on a huge number of samples will be addressed to the developed calibration models for the identification and quantification of the main bioactive compounds present in the waste.

The qualitative-quantitative knowledge of fatty acids and condensed tannins obtained with destructive analysis techniques can represent a first data-base useful to validate and make reliable the FTIR technique as quality and titre monitoring of active principles in grape seeds.

## Figures and Tables

**Table 1:** Fat content (g/100 g) and fatty acid profiles (percent of total fatty acid content). The values in parentheses represent the standard deviation.

Compound	Nero d’Avola	Cesanese d’ Affile	Greco Bianco
Fat content	8.66 (0.23) a	13.65 (0.71) b	8.06 (0.23) a
C12:0	0.24 (0.11) b	0.08 (0.03) ab	0.05 (0.02) a
C14:0	0.30 (0.09) a	0.19 (0.06) a	0.15 (0.05) a
C16:0	11.55 (1.18) a	12.19 (1.75) a	10.37 (0.72) a
C16:1	0.39 (0.17) a	0.25 (0.04) a	0.43 (0.07) a
C17:0	0.14 (0.03) a	0.12 (0.05) a	0.10 (0.03) a
C18:0	4.29 (0.21) a	5.01 (0.36) b	4.44 (0.09) ab
C18:1	23.09 (0.74) c	17.87 (0.11) a	21.07 (0.50) b
C18:2 ω-6	59.02 (0.97) a	63.71 (2.18) b	62.48 (1.68) ab
C18:3 ω-3	1.04 (0.19) a	0.69 (0.09) a	0.91 (0.23) a
C20:0	0.40 (0.15) c	n.d. *a	0.19 (0.13) b

Means within the same row with different superscripts letters (a, b and c) are significantly different (*p* < 0.05). * not detectable.

**Table 2:** HPLC/DAD/MS data expressed in mg/g of selected phenolic compounds present in the grape seeds. All results reported are the average of three replications and the relative standard deviation is less than 0.05.

Compound	Nero d’Avola	Cesanese d’ Affile	Greco Bianco
gallic acid	0.04 (0.00) a	0.15 (0.01) c	0.11 (0.00) b
catechin dimer B3	1.32 (0.00) a	2.81 (0.01) c	1.91 (0.01) b
catechin	1.77 (0.01) a	4.89 (0.01) c	3.09 (0.08) b
procyanidin trimer	0.00 (0.00) a	0.88 (0.03) c	0.48 (0.01) b
catechin dimer B6	1.33 (0.01) b	1.62 (0.05) c	0.89 (0.02) a
catechin dimer B2	0.78 (0.01) a	1.86 (0.01) c	0.98 (0.01) b
epicatechin	0.60 (0.00) a	3.63 (0.03) c	2.01 (0.01) b
catechin trimer	0.41 (0.02) a	2.01 (0.01) c	1.15 (0.02) b
epicatechin gallate dimer	1.66 (0.01) a	5.46 (0.03) c	3.46 (0.07) b
catechin oligomers expressed as tetramers	24.80 (0.61) b	0.62 (0.02) a	0.29 (0.01) a
epicatechin gallate dimer	17.30 (0.41) b	1.16 (0.06) a	0.61 (0.01) a
catechin/epicatechin trimers digallate	30.90 (1.10) c	11.17 (0.08) b	9.17 (0.15) a
catechin/epicatechin trimers gallate	5.01 (0.02) a	21.53 (0.20) b	22.53 (0.24) c
total	85.92	57.80	46.68

Means within the same row with different superscripts letters (a, b and c) are significantly different (*p* < 0.05).
